# In vitro nanoparticle dosimetry for adherent growing cell monolayers covering bottom and lateral walls

**DOI:** 10.1186/s12989-018-0278-9

**Published:** 2018-10-30

**Authors:** Linda Böhmert, Laura König, Holger Sieg, Dajana Lichtenstein, Niklas Paul, Albert Braeuning, Andreas Voigt, Alfonso Lampen

**Affiliations:** 1 Department of Food Safety, German Federal Institute for Risk Assessment, Max-Dohrn-Str. 8-10, 10589 Berlin, Germany; 2 Chair of Process Systems Engineering, Otto-von-Guericke University Magdeburg, Universitätsplatz 2, 39106 Magdeburg, Germany; 3 Technische Universität Berlin, Fachgebiet Verfahrenstechnik, Ackerstraße 71-76, 13355 Berlin, Germany

**Keywords:** Dosimetry models, Silver nanoparticles, Differentiated Caco-2 cells, In vitro

## Abstract

**Background:**

Even though a continuously high number of in vitro studies on nanoparticles are being published, the issue of correct dose matter is often not sufficiently taken into account. Due to their size, the diffusion of nanoparticles is slower, as compared to soluble chemicals, and they sediment slowly. Therefore, the administered dose of particles in in vitro experiments is not necessarily the same (effective) dose that comes into contact with the cellular system. This can lead to misinterpretations of experimental toxic effects and disturbs the meaningfulness of in vitro studies. In silico calculations of the effective nanoparticle dose can help circumventing this problem.

**Results:**

This study addresses more complex in vitro models like the human intestinal cell line Caco-2 or the human liver cell line HepaRG, which need to be differentiated over a few weeks to reach their full complexity. During the differentiation time the cells grow up the wall of the cell culture dishes and therefore a three-dimensional-based in silico model of the nanoparticle dose was developed to calculate the administered dose received by different cell populations at the bottom and the walls of the culture dish. Moreover, the model can perform calculations based on the hydrodynamic diameter which is measured by light scattering methods, or based on the diffusion coefficient measured by nanoparticle tracking analysis (NTA). This 3DSDD (3D-sedimentation-diffusion-dosimetry) model was experimentally verified against existing dosimetry models and was applied to differentiated Caco-2 cells incubated with silver nanoparticles.

**Conclusions:**

The 3DSDD accounts for the 3D distribution of cells in in vitro cell culture dishes and is therefore suitable for differentiated cells. To encourage the use of dosimetry calculating software, our model can be downloaded from the supporting information.

**Electronic supplementary material:**

The online version of this article (10.1186/s12989-018-0278-9) contains supplementary material, which is available to authorized users.

## Background

Growing numbers of nanoparticles are being developed and used for technical, research, food-related and medical applications. Their huge physicochemical variety poses a challenge for understanding their biological behavior and toxicological potential. To reduce the number of animals that are needed for toxicological assessment of nanoparticles, a number of read-across and grouping approaches, as well as numerous in vitro approaches to understand the mechanisms of uptake and toxicity have been developed [4, 11, 33, 38, 42]. In vitro experiments require the correct determination of nanoparticle dosing which is not necessarily equal to the administered dose [21, 36]. This is due to the fact that the size of nanoparticles has a major influence on their diffusion and sedimentation behavior, thus leading to an inhomogeneous distribution of nanoparticles in in vitro systems. Therefore, the delivered dose which reaches the cells to trigger biological effects is often unknown. This delivered dose can be calculated using in silico models, a small number of which have been published since 2010 [7, 13, 30, 32]. All models calculate the diffusion and sedimentation of nanoparticles in vitro*,* and some of them also account for aggregation and ion release [7, 13, 30, 32]. Although the number of in vitro studies related to nanoparticle toxicity is still rising, in silico calculations have been used in only very few studies to adjust the dose to the delivered dose. Moreover, not all published models are available to the research community.

Considerations about the correct dose are more popular in the field of inhalative in vivo toxicity, as some model calculations exist for the distribution of fine particulate matter in the respiratory tract [9, 23]. However, the calculation of the delivered dose is often not taken into account in work that deals with corresponding in vitro systems, such as lung epithelial cell lines. This problem increases when in vitro systems for intestinal or liver cells are used. Some of these cell lines are typically used in a differentiated state after several weeks of growth and differentiation. The most common in vitro model for the intestinal epithelium is the human cell line Caco-2. After reaching confluency, Caco-2 monolayers differentiate within 21 days to an enterocyte-like monolayer expressing several morphological and functional characteristics of a mature enterocyte, such as monolayer growth, a cylindrical polarized morphology with microvilli on the apical side, the formation of tight junctions between adjacent cells, and the expression of small intestinal hydrolase enzyme activities on the apical membrane [5, 37]. The importance of the Caco-2 cell model as a commonly and frequently used in vitro model for the intestinal barrier is also elucidated by the number of publications (970 hits in PubMed for nanoparticle and Caco-2, as per 17.09.2018). With regard to the induction of differentiation over a longer in vitro cultivation period, the cell line HepaRG constitutes a comparable model for hepatocytes and primitive biliary epithelial cells [12, 34].

In the course of previous work with Caco-2 cells, we noticed the tendency of these cells to grow up the walls: during the 3 weeks of differentiation, Caco-2 cells do not only form a confluent monolayer on the bottom of the cell culture dish, but also start growing up the encircling wall of the cell culture dish. Even though the cells start to grow as monolayer, they do not entirely stop cell division after getting confluent and therefore push the cell monolayer up the walls of the cell culture dish during the differentiation phase. This also applies to other differentiated cell culture models such as HepaRG (own unpublished observation). The coverage of bottom and walls of a cell culture dish is therefore especially relevant for dosimetric calculations of nanoparticles in experiments based on such differentiated cell monolayers. The proportion of cells growing not on the bottom but on the wall of the cell culture dish can account for a major fraction of cells especially in smaller formats such as 96-well plates and is, due to the diffusion/sedimentation properties of nanoparticles, expected to receive a delivered dose different from the delivered dose relevant for cells growing on the bottom of the plate. This 3-dimensional distribution of cells has been ignored so far in calculations of the effective dose of nanoparticles using the previously available models. Hence, we identified the need to adapt dosimetric calculations for the 3-dimensional distribution of cells in different cell culture formats. Therefore, a new, 3-dimensional model for calculation of the delivered dose is needed in order to account for differentiated cell models like Caco-2. Application of this model is not limited to differentiated Caco-2 cells with their special intestinal-like properties, but designed to contribute to dosimetric calculations also for other differentiated cell systems which share the ability to also populate the wall of a cell culture vessel.

The aim of this study was to develop an in silico model to calculate the nanoparticle distribution and dosimetry in all three dimensions to enable a proper interpretation of the results of toxicological studies with differentiated in vitro models. Moreover, we aimed at developing a 3D-dose-model capable of calculating the particle distribution on the basis of the diffusion coefficient measured by Nanoparticle Tracking Analysis (NTA), because NTA can measure particle movements directly and in the cell culture medium that is used for the in vitro studies. Besides, the new model should also be applicable for using the measured hydrodynamic radius, as previous models. The novel 3D-sedimentation-diffusion-dosimetry (3DSDD) model is assessed against experimental results and available published models. The 3DSDD model is freely available by download from the supporting information of this paper.

## Results

### Characterization of the Caco-2 model

**Fig. 1:**
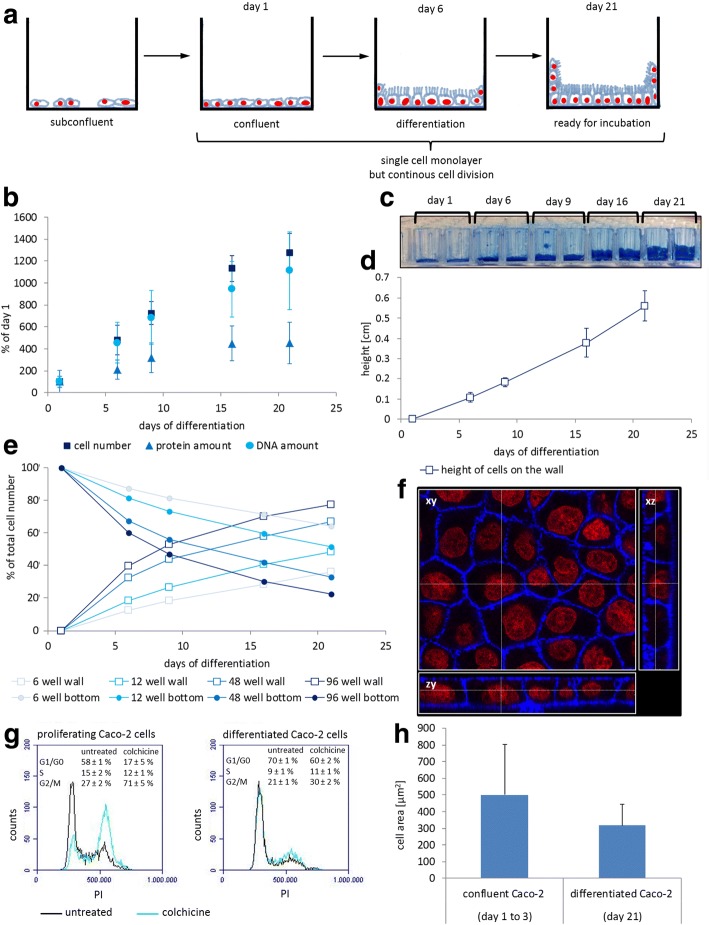
Characterization of the distribution of Caco-2 cells during differentiation. (**a**) Schematic overview of the distribution of Caco-2 cells in cell culture dishes during differentiation. (**b** to **e**) Characterization of Caco-2 cell differentiation in different cell culture plate formats concerning their cell number, protein and DNA content, and height of cell growth on the cell culture dish wall. Day 1 corresponds to the day the cells reached confluency. (**b**) Cell number, protein and DNA amount per 96-well plate well during differentiation. Mean and standard deviation of ≥3 replicates are shown. Cell numbers were analyzed after trypsin treatment using trypan blue staining and a Neubauer chamber. Protein amounts were determined by the Bradford Assay. DNA content was determined photometrically following phenol-chloroform extraction. Mean and standard deviation of ≥3 replicates are shown. (**c**) Cross section of a 96-well plate with coomassie-stained Caco-2 cells after 1, 6, 9, 16 and 21 days of differentiation. (**d**) Height of cell growth on the wall of culture dishes during Caco-2 differentiation. Height of cells was measured after coomassie staining and cutting the well plate in half with a sliding caliper. (**e**) Distribution of cells between bottom and wall of 6-well, 12-well, 48-well and 96-well plates during differentiation of Caco-2 cells. (**f**) Confocal microscopic image of a Caco-2 cell monolayer differentiated for 21 days. Nuclei (red, ToPro-3) and actin cytoskeleton (blue, ActinRed) were stained showing a confluent monolayer. (**g**) Cell cycle analysis of proliferating and differentiated Caco-2 cells. Additionally, cells were treated with colchicine to induce cell cycle arrest in the G2/M phase. Cell cycle was measured by flow cytometry after PI staining. Representative results are given in the diagram as counts over peak area and results of at least three different independent experiments are summarized in the numbers. (**h**) Analysis of cell size of confluent but undifferentiated and differentiated Caco-2 cells. Cell size was measured by image analysis of confocal microscopic images of actin-stained cells. Results from analysis of 10 images with at least 100 cells per image are shown

**Fig. 2:**
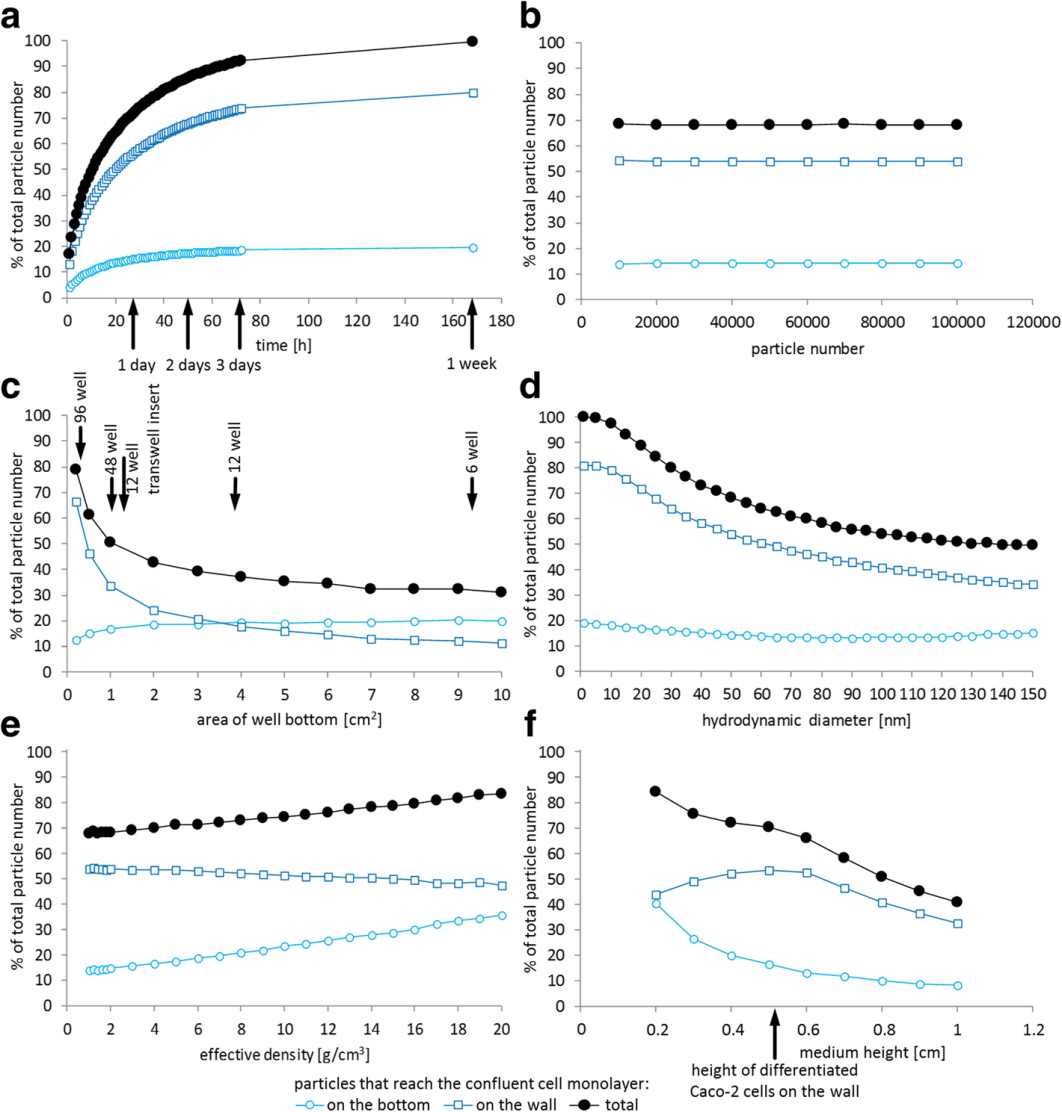
Impact of different parameters on the calculation of the delivered dose by the in silico dosimetry model introduced in this study (3D-sedimentation-diffusion-dosimetry, 3DSDD), applied to the cell growth of differentiated Caco-2 cells. Graphs show the fraction of particles that reach the differentiated Caco-2 cell monolayer on the bottom and wall of the cell culture vessel. The total fraction of particles reaching cells is also presented. In silico calculations were repeated three times and mean and standard deviation (too small to be visible in the graphs) were calculated. Results are given as percentage of the initial particle number. The following parameters were used for calculation: particle diameter 50 nm, effective density of nanoparticles 1.5 g/cm^3^, particle number 10000, temperature 37 °C, density of cell culture medium 1.0037 g/cm^3^, viscosity of cell culture medium 0.725 mPa s, height of cell growth on the cell culture dish wall 0.54 cm, surface area of cell culture dish bottom 0.34 cm^2^ (96-well plate), geometrical shape of cell culture dish is a cylinder, height of medium level in well 0.57 cm (corresponds to about 200 μL in 96-well, 570 μL in 48-well, 2.2 mL in 12-well and 5.5 mL in 6-well) and incubation time 24 h. In each diagram one parameter was altered: (**a**) time, (**b**) particle number, (**c**) area of well bottom, (**d**) hydrodynamic diameter of particles, (**e**) effective density of particles, (**f**) medium height

Caco-2 cells spontaneously differentiate into a confluent monolayer with typical morphological and biochemical features. Successful differentiation of Caco-2 cells into a confluent polarized monolayer has been shown in previous studies from our laboratory by proteomic analysis and mRNA expression profiling of intestinal markers [5], as well as by electron microscopy of cellular morphology [26, 27]. In addition to this functional characterization of Caco-2 cells, we now in-depth characterized parameters such as the cell number throughout the differentiation process. Our special focus was on fully differentiated Caco-2 cells in order to reveal the exact properties of this cell model for usage in dosimetric calculations for nanoparticles in vitro. Therefore, Caco-2 cells were characterized throughout their differentiation in various cell culture formats. As schematically shown in Fig. 1a, day 1 was set to the day where cells reached confluency. On days 1, 7, 9, 16 and 21, the cell numbers as well as DNA and protein contents were quantified (Fig. 1b). Even though Caco-2 cells grow as a strict monolayer as shown in Fig. 1f, and even though day 1 was defined as the day of reaching confluency, cell numbers continued to rise over the whole observation time. This was supported by the rising DNA content, whereas the protein content reached a plateau (Fig. 2b). Based on these observations, the question of the localization of the additional cells arose, as still only a monolayer of Caco-2 cells was present at the bottom of the culture vessel. Additional analyses of cell cycle (Fig. 1g) and cell size (Fig. 1h) showed an increase in G1/G0 phase cells and a reduced susceptibility towards colchicine, as well as a reduction of cell size or growth area per cell. Moreover, and most relevant for dosimetric calculations, we observed that the Caco-2 monolayer grew up the lateral wall of the cell culture dish during differentiation. This was shown by coomassie staining as presented in Fig. 1c. Cell growth at the lateral walls of the culture dishes was subsequently monitored in more detail: on day 21 cells reached a height of about 5.7 mm at the cell culture dish wall (Fig. 1c-d). The height of cell culture medium during differentiation was higher, e.g. 8.6 mm with 300 μL medium in a 96-well cavity. When differentiating between cells on the well bottom and at the wall, it can be seen that the cells on the wall can get more numerous than the cells on the bottom during differentiation depending on the cell culture format (Fig. 1e). The cell numbers on the wall continuously grew over time, like the height of cells on the wall (Fig. 1c-e). The increased cell numbers and DNA contents (Fig. 1b) are thus to be attributed, to a major part, to cells growing vertically on the wall of the cell culture dish. As shown in Fig. 1e, all other cell culture formats, like 48-, 12- and 6-well plates showed similar behavior, with somewhat differing percentages of cells on the bottom and wall due to the differences between these formats in the ratios of surface areas.

### Characterization of the in silico 3DSDD model

The delivered dose was calculated for a virtual nanoparticle with a particle diameter of 50 nm, an effective density of 1.5 g/cm^3^ and a particle number of 10,000. Typical cell culture conditions were used with a temperature of 37 °C, a density of cell culture medium of 1.0037 g/cm^3^, and a viscosity of cell culture medium of 0.725 mPa s (this corresponds to the measured density of DMEM with 10% FCS and 1% P/S as used for cultivating Caco-2 cells), a height of cell growth on the cell culture dish wall of 0.54 cm (measured cell growth on the wall for 21 days differentiated Caco-2 cells), a surface area of the cell culture dish bottom of 0.34 cm^2^ with a geometrical shape of a cylinder (96-well plate), 0.57 cm height of the medium level in the well (corresponds to about 200 μL in 96-well, 570 μL in 48-well, 2.2 mL in 12-well, and 5.5 mL in 6-well format), and an incubation time of 24 h.

Figure 2 gives an overview about the impact of different parameters on the results of the in silico calculations of the delivered dose to Caco-2 cells on the bottom and wall, as well as the total cell surface using the 3DSDD model as described above. A time-dependent increase in particle numbers that reach the cells can be seen, with a greater proportion of particles that come into contact with the cells on the wall of the cell culture dish (Fig. 2a). Figure 2b shows that a model calculation with 10,000 particles is as representative as calculating more particles while saving computation time. The influence of the cell culture dish format is depicted in Fig. 2c and correlates mainly with the ratios of wall and bottom as well as dish surface and volume. The influence of the nanoparticle-specific parameters hydrodynamic diameter and effective density is given in Fig. 2d and e: a bigger hydrodynamic diameter causes a reduction of the particle number that reaches the wall, whereas the particle number that reaches the bottom is slightly increasing. Nevertheless the fraction of the administered dose that comes in contact with cells is decreasing with an increasing hydrodynamic diameter. Figure 2f shows the influence of the incubation volume given as medium height. For rising particle dispersion volumes the percentage of particles that come in contact with cells is decreasing, except for heights of the particle dispersion that are lower than the cells height on the cell culture dish wall (0.54 cm). When the incubation volume is further increased to raise the medium level in the culture vessel above the level of cell growth on the wall, no additional cells are exposed and the percentage of particle that reaches cells shows the same relative decrease as the percentage of particles that reach the cells on the bottom.

The dissolution of ions from particles in cell culture media has been discussed by Thomas et al. in great detail [41] and might affect particle properties and thus also impact on dosimetric calculations. Silver ion release of the two particles used in our study corresponded to a mass dissolution of about 2 to 6% of the total particle mass in cell culture medium after 24 h (determined experimentally and given in Fig. 5). This would result in only very small changes to the particles: it can be calculated that the above mass loss of a particle corresponds to an only very minor decrease of the size of a hypothetical 20 nm diameter particle core down to 19.4 nm (i.e., an only 3% decrease of the particle diameter). Furthermore, based on the amount of silver released from the particles and based on our model calculations, the amount of material delivered to the lateral wall or to the bottom by ion diffusion will change the total amount of delivered silver by only 1% or less. We thus conclude that the contribution of released silver ions is very minor and therefore is only considered in the last part of our study (use of the in silico 3DSDD model for Caco-2-based experiments).

**Fig. 3:**
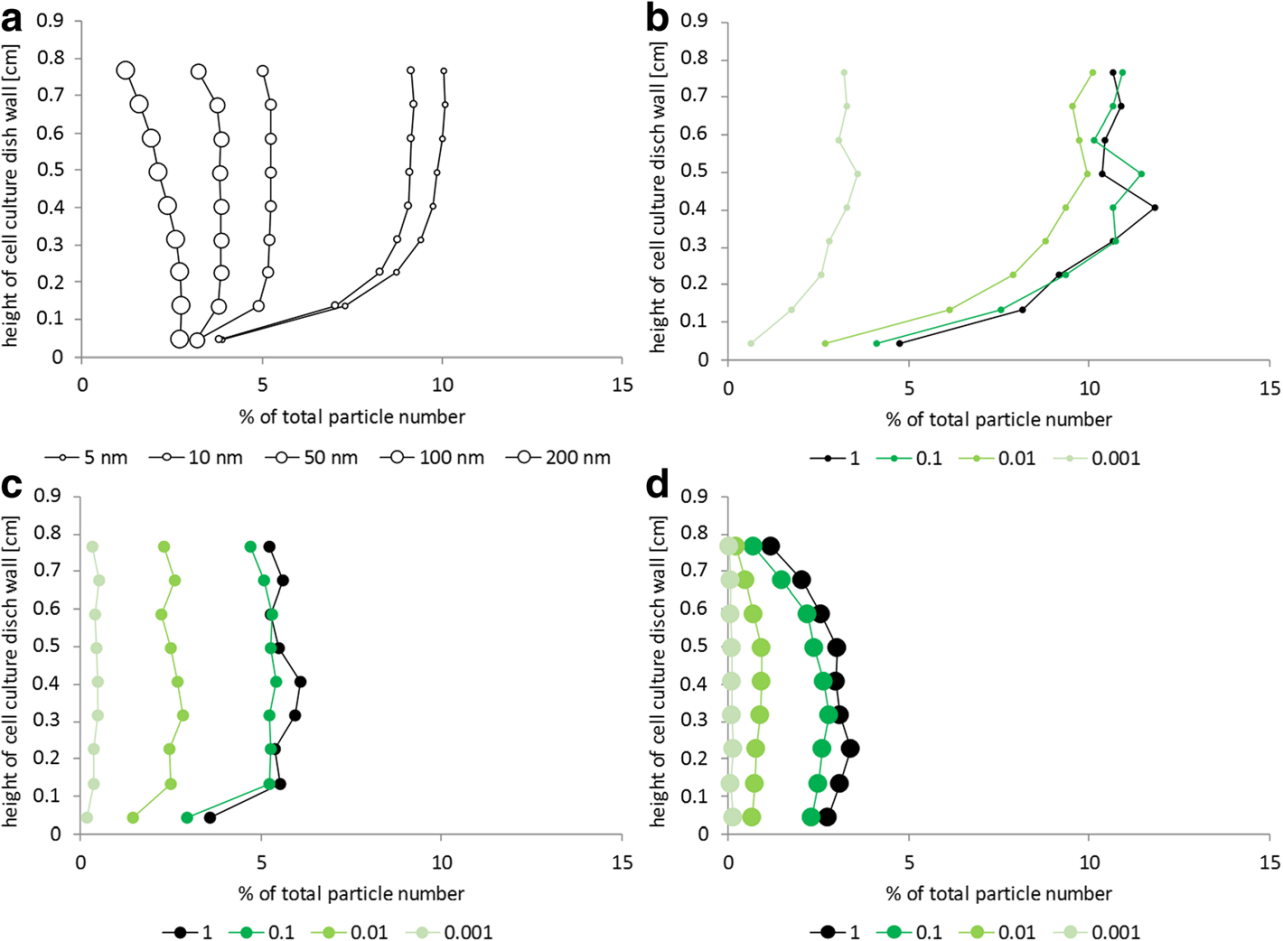
Impact of particle size on the spatial distribution of particles on the wall disregarding cell growth using the 3D-sedimentation-diffusion-dosimetry (3DSDD) model. Graphs show the fraction of particles that reach the wall of the cell culture well. The following parameters were used for calculation: effective density of nanoparticles 1.5 g/cm^**3**^, particle number 10000, temperature 37 °C, density of cell culture medium 1.0037 g/cm^3^, viscosity of cell culture medium 0.725 mPa s, surface area of cell culture dish bottom 0.34 cm^**2**^ (96-well plate), geometrical shape of cell culture dish is a cylinder, height of medium level in well 0.88 cm and incubation time 48 h. In each diagram one parameter was altered: (**a**) The particle number distribution in percent of total particle number is given for the different particle diameters 5, 10, 50, 100 and 200 nm. (**b** to **d**) Similar calculations are presented for 5 nm particles (**b**), for 50 nm particles (**c**) and for 200 nm particles (**d**) dependent on variations of the sticking coefficient (see text for details): c_stick_ = 1, c_stick_ = 0.1, c_stick_ = 0.01 and c_stick_ = 0.001

The height distribution of particles delivered to cells growing on the wall is of interest, especially with respect to different particle sizes. Particles, in the beginning distributed evenly in the medium, will diffuse and sediment. According to our 3DSDD model, they can stick to the upper part of the wall, but also to lower parts. The total amount of particles interacting with the wall is remarkable, as can be seen in Fig. 2d. Figure 3a shows that for small particles which do not sediment quickly to the bottom, a higher percentage will stick to the upper parts of the wall. Overall, for 5 nm diameter particles more than 50% of the total applied amount sticks to the upper part of the wall. For larger particles the overall percentage decreases, as well as the percentage of particles that is present in dispersion in the upper layers of the well. The biggest model particles with a diameter of 200 nm will quickly sediment to the bottom; only a small percentage will stick to the wall, in particular to its lower parts. Our 3DSDD model predicts the effect of spatial distribution inside a well quite nicely and a first estimation on the effect of wall-grown cells and the delivered dosage will be important for further steps of dosage estimation and evaluation. These example calculations demonstrate that for particles with different sizes the delivered cellular doses differ between cells on the bottom and on different positions on the wall. Differences in sedimentation and diffusion deliver bigger particles faster to the bottom, as compared to smaller particles, which have more opportunities to interact with cells on the wall.

For the above calculations we have initially assumed that in case a particle hits the wall (or a cell growing on the wall) once, it will stick to it (i.e., it will be counted as delivered to the cell). In reality not all wall-particle interactions will lead to an immediate sticking. We therefore introduced sticking efficiency as a parameter in our model and used it to investigate the effect of stickiness variation on the height distribution of particles delivered to the wall in more detail (Fig. 3). The effect of particles sticking to the wall was investigated in a simplified 3D in silico simulation without cells on the wall. The initial stickiness, where each interactions leads to sticking to the wall, corresponds to a value of c_stick_ = 1 for the stickiness coefficient. The stickiness parameter was varied from 1 to 0.001 for three different model particle sizes (5, 50 and 200 nm) chosen to reflect typical sizes of nanoparticles used in experimental in vitro studies. For the smallest particle (5 nm), calculations revealed that an effect of a reduced stickiness factor is only observable for a strongly reduced coefficient of 0.001. This number means that only 1 of 1000 collisions of a nanoparticle with the wall leads to sticking of the particle to the wall. For the other evaluated c_stick_ values ≥0.01, the numbers of particles sticking to the wall were almost the same for the 5 nm particle (Fig. 3b). The lowest part of the wall does not often receive hits by particles due to a depletion effect on the particle density over time. For 50 nm particles the number of particles sticking to the wall was smaller (Fig. 3c). Here, a reduction of stickiness to c_stick_ = 0.01 already led to a notable change in the number of particles sticking to the wall. The same was calculated for 200 nm particles (Fig. 3d). For further analyses performed in the course of the present study we kept the stickiness factor constant at c_stick_ = 1 for all cell-covered surfaces, because the above results show that only strongly reduced stickiness factors may have the potential to remarkably change the resulting data obtained from the calculations.

### Verification of the in silico 3DSDD model against other models

For verification, our 3DSDD model was compared to different existing models from the literature using particles and their characteristics published in the corresponding papers describing these models. First, we used the volumetric centrifugation method (VCM)-modified ISDD model from Hinderliter et al. and DeLoid et al. [6, 13]. CeO_2_ and gold nanoparticles and their characteristics were taken from DeLoid et al. and are listed in Table 1 [6]. Second, we used the DG model from DeLoid et al. [7] and TiO_2_ and SiO_2_ nanoparticle characteristics from the same study (cp. Table 1). These particles were chosen to reflect the complete spectrum of particle size and effective density given in these two publications.

**Table 1:** Parameters used for comparing the DG and VCM-ISDD models with the 3DSDD model

	DG		VCM-ISDD	
particle type	titanium dioxide	silicon dioxide	cerium dioxide	gold
hydrodynamic diameter [nm]	397.8	149.9	131	42.2
effective density [g/cm^3^]	1.251	1.564	2.368	17.73
Particle number [#]	10,000	10,000	10,000	10,000
temperature [°C]	37	37	37	37
medium density [g/cm^3^]	1.0104	1.0104	1	1
medium viscosity [mPa s]	0.81	0.81	0.74	0.74
filling level of the dish [cm]	0.3	0.3	0.315	0.315
simulation time [h]	72	72	72	72
reference	[7]	[7]	[6]	[6]

**Fig. 4:**
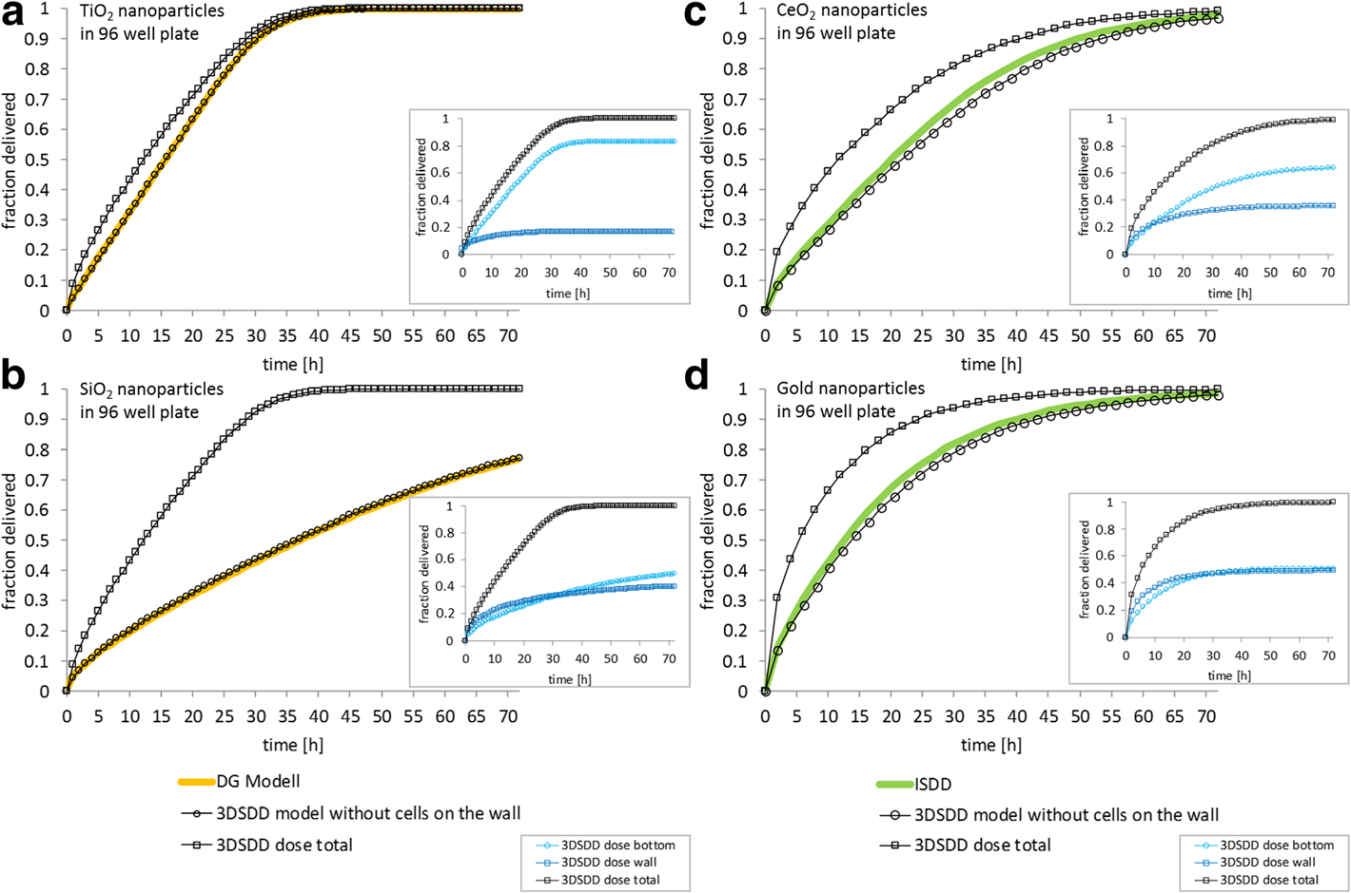
Verification of the 3DSDD model by comparison with the published models DG and volumetric centrifugation method (VCM)-ISDD for nanoparticle incubations of up to 3 days. (**a** to **b**) The DG model was used to calculate the delivered dose of TiO_2_ and SiO_2_ nanoparticles using parameters from DeLoid et al. [7]. (**c** to **d**) The VCM-modified ISDD model was used to calculate the delivered dose of CeO_2_ and gold nanoparticles using parameters from DeLoid et al. [6]. The parameters used for calculation are given in Table 1. The same parameters were used for calculating the delivered dose with the 3DSDD model. For proper comparison 3DSDD calculations were done for cell on the bottom only, like in the DG/VCM-ISDD. In addition, 3DSDD results were calculated for differentiated Caco-2 cells growing on bottom and wall of the cell culture dish; these data are also shown in the graphs to visualize the impact of vertically growing cells on the total delivered dose. The inserts show the effective nanoparticle doses calculated with the 3DSDD model for differentiated Caco-2 cells as a subdivision into both fractions, cells on the bottom and cells on the wall of the cell culture dish. Abbreviations: 3DSDD: 3D-sedimentation-diffusion-dosimetry model; VCM: volumetric centrifugation method used for determining the effective density of nanoparticle dispersions; ISDD: In vitro Sedimentation, Diffusion and Dosimetry model; DG: Distorted Grid model

In Fig. 4a and b the DG model in silico results for TiO_2_ and SiO_2_ nanoparticles are given in comparison with the results from 3DSDD, either with the same parameters and without considering cells on the wall, or with cells on the cell culture dish wall. In Fig. 4c and d the same comparison is given for the VCM-modified ISDD model in silico results for CeO_2_ and gold nanoparticles are given. As these examples are only used to compare different in silico models an average particle size was used. Never the less, the 3DSDD model is also capable to calculate on the basis of particle size distribution like the DG model is. Moreover, it can use directly measured diffusion coefficient distribution data from NTA measurements. As shown in Fig. 4, the results from our 3DSDD model resemble the results of the two other models when calculating the dose with the same parameters and the assumption that the cells grow on the bottom only. However, for incubation of differentiated cell culture systems that grow up the wall, there is a slightly higher amount of particles that reach the cells during the first 30 h of incubation. This difference is due to the amount of particles that reach the cells on the wall of the cell culture dish. Additionally, the cells on the bottom of the cell culture dish do not come in contact with all cell-contacting nanoparticles, as some of them interact only with the cells on the wall.

### Verification of the in silico 3DSDD model against experimental data

**Fig. 5:**
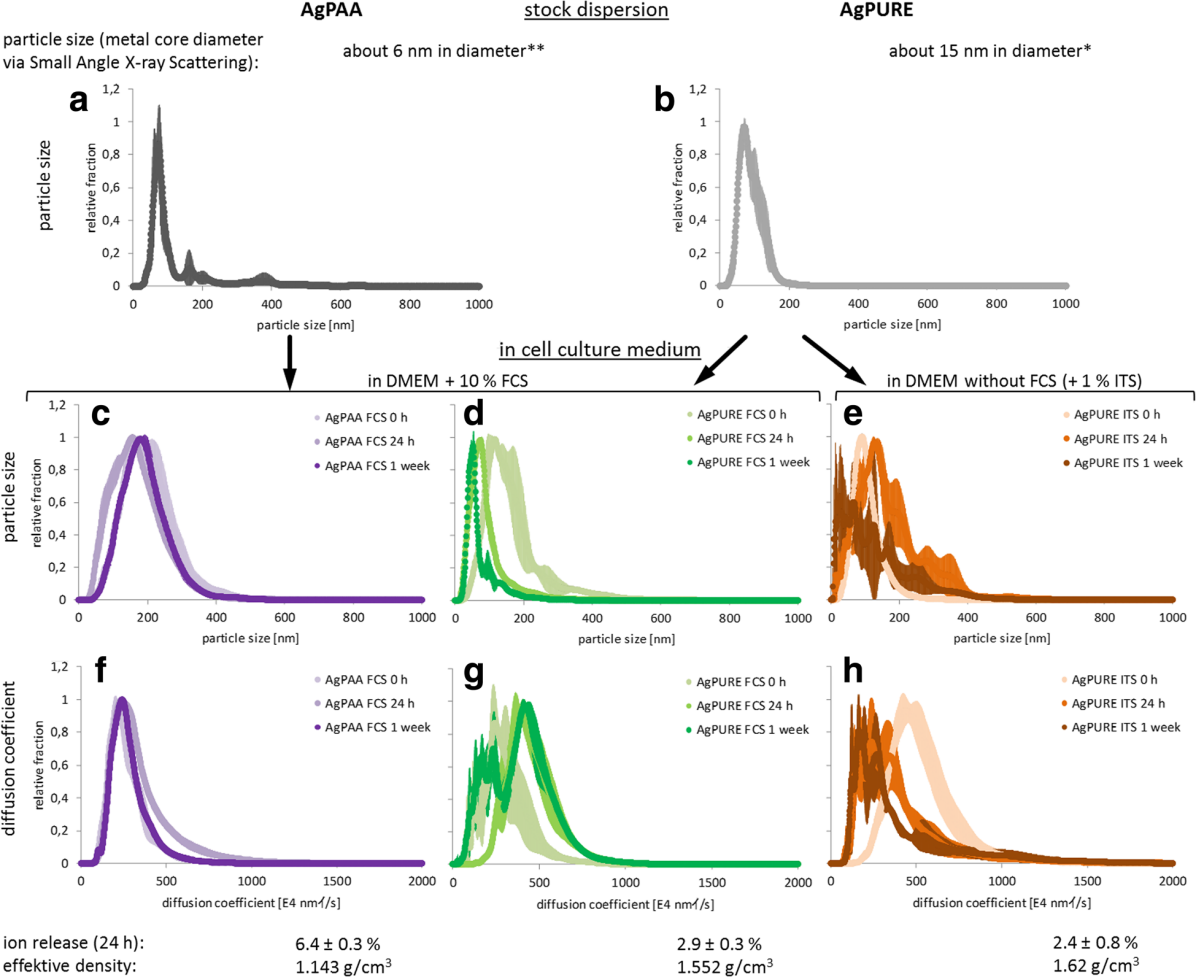
Overview and characterization of AgPAA and AgPURE silver nanoparticles. (**a** and **b**) Representative measurement of particle size distributions of AgPAA and AgPURE in stock dispersion by Nanoparticle Tracking Analysis (NTA). (**c**, **d** and **e**) NTA particle size measurement of AgPAA in serum-containing cell culture medium (DMEM + 10% FCS), AgPURE in serum-containing cell culture medium, and AgPURE in serum-free cell culture medium (DMEM + 1% insulin/transferrin/selenium (ITS)) after 0 h, 24 h and 1 week of incubation under cell culture conditions (37 °C, humidified atmosphere and 5% CO_2_). (**f**, **g** and **h**) NTA measurement of the diffusion coefficients of AgPAA and AgPURE under the above conditions. Asterisks indicate relevant previous publications where further details on the characterization of AgPAA and AgPURE nanoparticles have been published: *[3] **[18, 26, 27] (see also reference section of this paper)

Prior to the verification of our in silico model against experimental data, nanoparticles were chosen for experimentation and characterized in-depth, in addition to the measurement of the parameters needed for the model calculations as given in Table 2 in the methods section. Figure 5 gives an overview of the characteristics of two different silver nanoparticles in stock dispersion, as well as in dispersions of both particles in cell culture medium. On the one hand, we used a silver nanoparticle with a silver core diameter of about 6 nm coated with poly-acrylic acid (PAA), termed AgPAA. On the other hand, a second bigger nanoparticle with about 15 nm in its metal core diameter and coated with two surfactants was selected (AgPURE). Both particles were delivered in a stable stock dispersion as verified by periodic measurement of hydrodynamic particle sizes (Fig. 5a and b). These stock dispersions were freshly diluted in cell culture medium for all experiments. Figure 5c to e shows measurements of hydrodynamic particle diameters after 0 h, 24 h and 1 week of dispersion in DMEM cell culture medium with or without serum. Additionally, Fig. 5f to h shows the corresponding diffusion coefficients. These measurements demonstrate that AgPAA forms a very stable dispersion in serum-containing cell culture medium (10% FCS). The dispersion of AgPURE is somewhat less stable in serum-containing cell culture medium. This situation is representative for many toxicological studies because this particle is often used in serum-containing cell culture medium [4, 10, 19, 20, 31, 40]. An example of less stable nanoparticle dispersion is AgPURE in cell culture medium without serum. Ion release from the particles was also measured. Analyses by Atomic Absorption Spectroscopy (AAS) revealed the ion release to be 6.4 ± 0.3% for the small AgPAA in serum-containing medium, and 2.4 ± 0.8% to 2.9 ± 0.3% for the bigger particle AgPURE in the presence or absence of serum, respectively. The effective densities were 1.143 g/cm^3^ for AgPAA and 1.552 g/cm^3^ for AgPURE in cell culture medium with FCS, and 1.698 g/cm^3^ for AgPURE under serum-free conditions.

**Table 2:** Parameters that are necessary to calculate the delivered dose for the silver nanoparticles AgPAA and AgPURE in cell culture medium containing either 10% fetal calf serum (FCS) or 1% insulin, transferrin and selenium (ITS), using the 3DSDD model

	AgPAA FCS	AgPURE FCS	AgPURE ITS
effective density [g/cm^3^]	1.143	1.552	1.698
Particle number [#]	10,000	10,000	10,000
temperature [°C]	37	37	37
medium density [g/cm^3^]	1.0037	1.0037	1.0030
medium viscosity [mPa s]	0.725	0.725	0.703
filling level of the dish [cm]	0.88	0.88	0.88
height of cell growth on side wall [cm]	0.54	0.54	0.54
simulation time [h]	168	168	168
diameter of primary particles [nm]	6.8	13.8	13.8

Effective density, medium density and viscosity, and height of cell growth on the side wall were determined experimentally. The diffusion coefficient or hydrodynamic size distribution are not given in the table, as it was used as the complete distribution measured by NTA after 24 h (see Fig. 5)

**Fig. 6:**
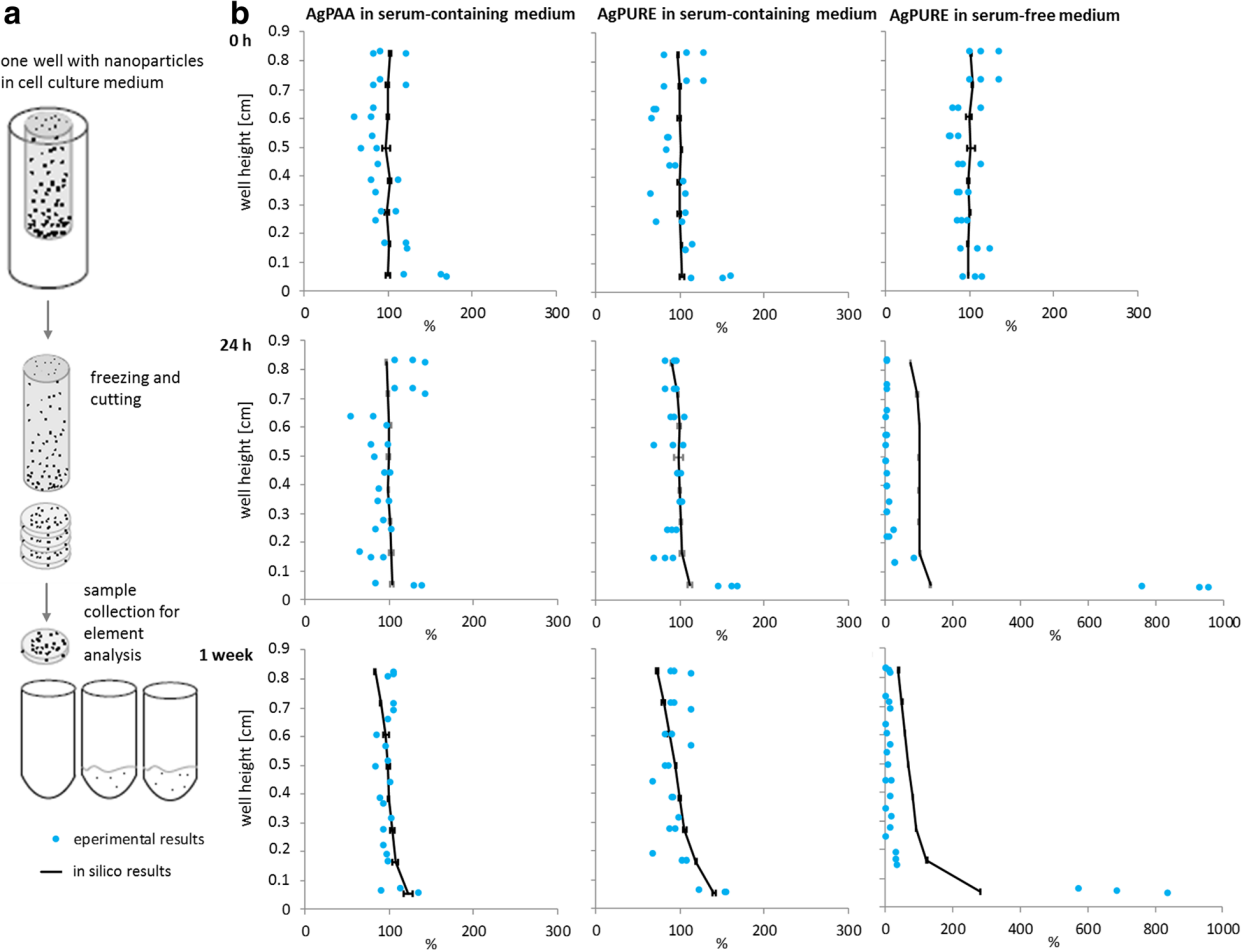
Verification of the 3DSDD model by element analysis in cryosections from cell culture medium. (**a**) Principle of the analysis. Nanoparticle dispersions in cell culture medium were incubated under cell culture conditions for 0 h, 24 h and 1 week in vessels with approximately the same size as 96-well plate cavities. After incubation, suspensions were snap-frozen at − 80 °C. Samples were then chilled to − 20 °C and cut into horizontal sections. The amount of silver in every section was analyzed by Atomic Absorption Spectrometry (AAS) after acidic digestion. (**b**) Comparison of the silver concentration profiles predicted by the 3DSDD model with the experimentally determined values for AgPAA and AgPURE in serum-containing cell culture medium and AgPURE in serum-free medium. The latter conditions in serum-free medium were chosen as an example of instable nanoparticle dispersion. The initial concentration was set to 100%

In addition to the model verification by comparison with other published models (Fig. 4), the 3DSDD model was now experimentally verified. For this purpose, cryosections were prepared from frozen cell culture medium columns from vessels of approximately 96-well size containing the above-characterized nanoparticles, incubated in different media as detailed above. Figure 6a contains a schematic delineation of the principle of this method. The serum-free condition was specifically chosen to represent unstable nanoparticle dispersion with a faster sedimentation rate. Element concentrations within the section were determined by AAS. The experimental results for the distribution of silver nanoparticles were obtained for incubation times of 0 h, 24 h, and one week. Results are given in Fig. 6b. The silver concentration profiles predicted by the 3DSDD model based on the diffusion coefficients measured at 24 h were in close agreement with the profiles obtained from the cryosection samples for AgPAA in serum-containing cell culture medium for all time points. An overall well agreement of in silico and experimental data appeared for AgPURE in serum-containing cell culture medium, with a slight underestimation of particle concentrations in the lowest section of the vessel by the model for shorter incubation times. These differences between experimental and in silico data aggravate for the even less stable particle dispersion of AgPURE in serum-free cell culture medium, where the model clearly underestimated the experimentally determined particle concentrations. Thus, less stable particles sediment faster than calculated by the model. This is likely to be due to the nature of particle characterization data: particle size measurements determine dispersed particles, whereas bigger agglomerates are formed from less stable particles which escape the measurement by their fast sedimentation.

### Use of the in silico 3DSDD model for Caco-2-based experiments

**Fig. 7:**
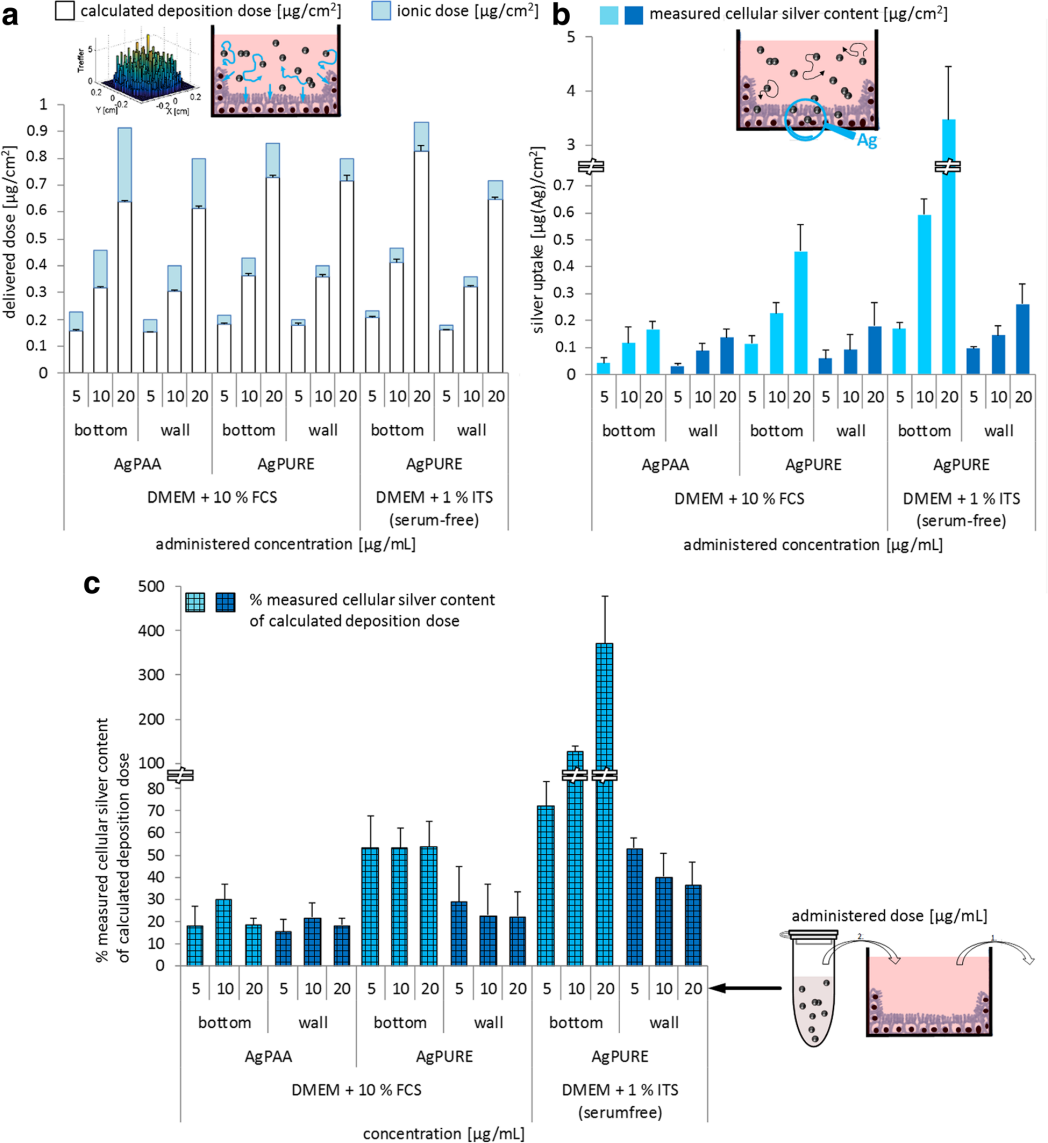
Correlation of the uptake of silver into differentiated Caco-2 cells on the bottom and wall of cell culture dishes after 24 h of incubation and comparison with the in silico-calculated delivered dose of silver nanoparticles. The incubation volume was 500 μL which equals a medium height of 4.5 mm in the insert. Therefore, 1.12 cm^**2**^ cell monolayer on the bottom and 1.688 cm^**2**^ cell monolayer on the wall are covered with cell culture medium. To determine the uptake of silver into the cells, we used element analysis by AAS subsequent to several washing steps aimed at the removal of particles loosely bound to the outside of the cells (see methods section for details). (**a**) Calculated delivered dose of the silver nanoparticles under serum-containing (DMEM + 10% FCS) and serum**-**free (DMEM + 1% ITS) cell culture conditions using the 3DSDD model. (**b**) Experimentally determined silver content of differentiated Caco-2 monolayers after 24 h of silver nanoparticle incubation. Loosely bound particles and ions were rinsed off prior to measurement of silver content and are thus not incorporated in the bars. (**c**) Experimentally determined silver amount from silver nanoparticles in Caco-2 cells, presented as the percentage of in silico-calculated delivered dose of the used particles. Mean and standard deviation are calculated from at least three replicates

In a next step the 3DSDD model was utilized to calculate effective in vitro nanoparticle doses as basis for the interpretation of silver uptake into Caco-2 cells. Therefore, we incubated the cells with the silver nanoparticles as characterized above and correlated the silver uptake (shown in Fig. 7b) with the in silico-calculated effective dose of silver nanoparticles (shown in Fig. 7a). Cellular silver contents were determined in a way that several washing steps were conducted prior to element analysis by AAS, in order to rinse off non-ingested, loosely bound particles from the cell surface. The data thus represent the particles taken up by or tightly bound to the cells, whereas particles which have been delivered to the cells but show only weak interactions with the cell surface are not included. Both, experimental results and in silico calculations are separately presented for cells on the bottom of the cell culture well and cells on the wall of the cell culture well. Caco-2 cells on the bottom are confronted with diffusing and sedimenting particles, whereas Caco-2 cells on the wall are just confronted with diffusing particles, affecting the delivery of nanoparticles to the cells. Experimentally determined uptake of particles is presented as the percentage of the calculated uptake during the incubation period (Fig. 7c).

For all three incubation conditions (AgPAA in serum-containing medium, AgPURE in serum-containing medium, AgPURE in serum-free medium) comparable delivered nanoparticle doses were calculated. Slightly higher effective doses were calculated for cells on the bottom (Fig. 7a). The above cryosection experiment (Fig. 6) with AgPURE particles in serum-free medium has shown that the model tends to underestimate the delivered dose at the bottom in case of instable particle dispersions forming fastly-precipitating aggregates. For AgPAA and AgPURE particles in serum-containing medium, the ratio of the calculated delivered dose and the experimentally measured cellular silver content is more or less identical for all three administered concentrations (Fig. 7c). For AgPAA silver nanoparticles the measured silver uptake per area of cell monolayer is the same for wall and bottom of the cell culture dish, whereas for AgPURE it is not. The comparison of the 3DSDD model results and cryosection experiments with AgPAA (Fig. 6) revealed the best correlation. Particle size measurements at different time points (Fig. 5c) showed a very weak tendency of the latter particles to agglomerate and therefore AgPAA formed the most stable particle dispersion in cell culture medium within our study. This is also reflected in the particle uptake per cell monolayer surface (Fig. 7c) which is identical regardless the spatial orientation of the cells. This implies that sedimentation does not play a major role in case of the AgPAA particle and the chosen incubation conditions. AgPURE in serum-containing cell culture medium seems to interact more with the cells on the bottom of the cell culture dish than on the wall as can be seen in Fig. 7c. The dispersion stability of that particle in protein-rich medium is much better than without proteins. Particle size distribution analysis at different time points (Fig. 5d) revealed a changing particle size over time. This also influences the comparability of the cryosection experiments and model results (Fig. 6): there, the 3DSDD model slightly underestimates the effective dose delivered to the bottom of the well. This exemplifies the need for stable particle dispersions as a major limitation of in silico models.

## Discussion

We observed that Caco-2 cells grow in strict monolayer but keep on dividing and growing in cell number during their 3 weeks of differentiation even after having reached confluency. Caco-2 cells are a frequently used model for intestinal epithelial cells since decades, as they possess typical enterocyte-like properties [24, 25, 35]. Some studies have reported a correlation between G1/G0 arrest and Caco-2 differentiation, which is corroborated by our cell cycle analysis demonstrating an increased fraction of cells in the G1/G0 phase at the end of the differentiation phase [8]. However, the remaining presence of cells in the S and G2/M phases, as well as the susceptibility towards colchicine showed that the cell line is still dividing during differentiation. This will result in cells getting slimmer to fit better into a more and more compressed monolayer on the culture vessel bottom. Additionally, we demonstrated that the monolayer was pushing itself up the walls of the cell culture dish. Both changes during differentiation, i.e. the increasing number of cells at the bottom, as well as the fact that additional cells grow on the vertical walls of the culture dish, are of importance when nanoparticle dosimetry is to be calculated.

Here, we present a new mathematical model for dosimetric calculations of the delivered dose of nanoparticles in vitro. While many scientists agree on the importance of dosimetry in nanotoxicology, these models are still not frequently applied, which might be due to difficulties with their public availability or with the need to use computational tools not familiar to many experimental toxicologists. Previously existing models have shown their value in calculating the delivered dose of nanoparticles in vitro under different conditions [7, 13, 30, 32]. However, none of these models has so far considered the fact that the cells in a cavity of a multi-well plate, which are usually analyzed all together and treated as a single homogenously exposed population, can be subdivided into one sub-population growing on the bottom of the cell culture dish, and a second sub-population growing vertically on the surrounding wall of the culture dish. Data on vertical growth show that it begins shortly after cells have reached confluency. In turn this suggests that cells on the wall of the cell culture dish might not be of pivotal relevance for sub-confluent cultures of cells grown only for a short period of time, but become increasingly relevant with cultivation time. Due to the differences in bottom/wall area ratios, the proportion of cells growing at the wall is greater in small (e.g. 96-well) cell culture formats. Advanced in vitro testing increasingly involves sophisticated, long-term cell cultivation and differentiation techniques [17, 40], and modern technologies for high-throughput measurement of different types of endpoints tend to prefer rather small multi-well format plates [16], thus pointing towards continuously increasing relevance of this previously neglected cell population growing on the surrounding wall of the cell culture dish and not on its bottom. For example, this type of growth behavior is also observed for the popular liver cell line HepaRG that needs to be differentiated over 4 weeks.

Our model validation demonstrates that the new 3DSDD is equivalent to previously existing dosimetry models with regard to the prediction of the overall delivered dose [7, 13]. Moreover, our model allows separate predictions of the delivered dose to cell populations either growing on the bottom or on the wall of a cell culture plate. Therefore, it is usable for differentiated as well as undifferentiated cell models. Presented data obtained with different silver nanoparticles show that in silico calculations with the new 3DSDD can well predict the effective dose of nanoparticles in in vitro experiments when the particle dispersions are stable over time, and that these predictions correlate well with the experimentally determined uptake of silver into differentiated Caco-2 cells, thus underlining the usefulness and applicability of the model. The presented data also gives an example for the tendency of in silico models to underestimate the delivered dose for less or instable dispersions. This tendency is common to all in silico dosimetry models available at present and may lead to an overestimation of cellular particle uptake (because experimentally determined particle uptake is referred to an underestimated delivered dose). Such overestimations can occur for particle uptake based on element analysis in cellular fractions which is an often used experimental setup for uptake studies.

Another important area of application for in silico dosimetry calculations are particle uptake studies. For these investigations it is important to use particles doses that do not saturate the particle uptake mechanism of cells, to be sure that uptake is independent from the administered dose, as discussed by Hinderliter et al. for the interpretation of the results from Lison et al. concerning the cellular uptake of silica nanoparticles with respect to the dose [13, 29].

Another aspect is that our 3D model calculates the delivered dose for all cells in a differentiated cell model that contribute to the results from assays that measure an endpoint as the sum signal of all cells contained in one well of a multi-well plate, like for example most cell viability assays do. If differentiated Caco-2 cells, HepaRG cells, or other cell lines with comparable vertical growth are used in combination with nanoparticles that exhibit a pronounced tendency for sedimentation (such as e.g. the AgPURE particles in serum-free medium in our analyses), the delivered doses to the different cell subpopulations on the bottom or wall of the cell culture dish will be different. Thus, if sum signals are measured, especially in smaller cell culture formats where the growth area at the wall accounts for a substantial fraction of cells, these sum signals will reflect reality only in a kind of distorted way, as cell populations having received strikingly different nanoparticle amounts are lumped together. This might lead to misestimation of the degree of particle uptake or of the severity of a biological effect caused by the particles [28]. Of the currently available nanoparticle dosimetry models, the 3DSDD model is the only one to account for these differences between cell populations thus allowing for a more accurate estimation of delivered doses in such in vitro studies than possible with previously available models. Additionally, with respect to the particle characterization that is needed to calculate a delivered dose by in silico models, we expanded our input options from the hydrodynamic diameter of the nanoparticles to the diffusion coefficient itself. Thus, hydrodynamic diameters from light scattering methods can be used for calculations, as in other models, but also the diffusion coefficient measured by NTA [14].

It is true for every model that it only constitutes a simplified version of reality and therefore it is essential to know its applicability domain and limitations. Discrepancies between calculations by a model and experimental values can arise from factors which may not be accounted for by the model or which are not controlled by the experimental setup. From the present data it becomes clear that faster downward diffusion and sedimentation of bigger particles reduces the chance of wall hits and subsequent sticking, as expected. More importantly, our simulations show that stickiness may have some impact on the calculations by the 3DSDD model, even though changes to this parameter have to be major in size to provoke remarkable changes in the resulting simulation data. Therefore, the stickiness factor can be neglected for most calculations under standard conditions. Nevertheless this exercise shows that particle delivery to the cells depends on many factors and that a careful and thorough model investigation can help to elucidate important factors of particle delivery and dosage. In experimental setups with cells on the wall, the exact stickiness factor may depend on different parameters, for example the type of particle-cell interaction, the roughness of cells, and the local geometry of cell growth. Of note, the influence of stickiness at the bottom of cell culture dishes has already been discussed by DeLoid et al. for their DG model in comparison to the ISDD model. As the true binding properties between the particles and the cells are not known, DeLoid et al. used a Langmuir isotherm in order to introduce stickiness into the DG model and applied that approach to the well bottom by comparing different possible scenarios with a variable parameter K_D_. They came to the conclusion that, as the interaction of nanoparticle agglomerates with the cell surface is probably of a weak non-specific type, the effect of particle-cell binding on the transport is negligible in most cases [7]. At the bottom of the well the stickiness factor has not been introduced. Here, it can be expected that particles that do not stick to the bottom following the collision will nonetheless be located at or near to the bottom of the vessel at the end of the experiment as a consequence of particle sedimentation. Stickiness might influence the local distribution in some way, but only very low stickiness factors would have to be considered. We would nevertheless recommend analyzing that factor in more detail, because it might have an impact on specific local cell dosage and delivery for culture vessels with larger bottom areas or different well geometry. In principle, such an effect could be easily incorporated in our model by adding a factor.

Apart from the model properties, results of model calculations can only be accurate for accurately characterized nanoparticle dispersions. Especially the particle size measurement in dispersion with NTA or DLS for fastly agglomerating and sedimenting particles may be a source of inaccuracy. Even a slow agglomeration or slightly instable particle dispersion may lead to an underestimation of the effective dose by the in silico model especially after longer incubation times, as compared to the situation in vitro. Moreover, particle dispersion stability may change during incubation time, which is not reflected by the model calculations. Therefore, a characterization of particles under the actually used cell culture medium conditions is essential.

As already mentioned above, our model tends to underestimate the delivered dose at the bottom in case of instable particles dispersions forming fastly-precipitating aggregates. Such changes in particle dispersion during the time are not reflected by our or other dosimetric models. For the instable particle dispersion of AgPURE in serum-free medium, data obtained from incubation with different particle doses show that the measured silver content in the cells on the bottom rises in a more pronounced way than expected based on the assumption of a rather linear correlation of administered and delivered dose. This leads to a cellular silver content of more than 100% of the model calculations. Both, the underestimation of the calculated effective dose and the exponential rise in cellular silver content highlight the need for stable nanoparticle dispersions for experimental setups to enable proper interpretation of the results. Nevertheless, compared to previous models, the 3DSDD model is capable to calculate effective doses for both, the cells on the bottom and the cells on the wall.

Also, particle dissolution may indeed have a significant impact on the biological effects of particle exposure. Complete particle dissolution in a short time is seldom, whereas a limited degree of ion release can be observed for a number of metal or metal oxide particles. Some recently published models account for the complex aspect of ion release. [41] The dissolution rate of silver nanoparticles is comparably small, and Thomas and co-workers found that the latter effect accounts for less than 5% of total delivery. We observed a 2 to 6% ion release from the silver nanoparticles used in our study. Thomas et al. have used citrate-coated silver nanoparticles with silver core diameters of 20 and 110 nm in serum-containing cell culture medium. The silver ion release rate in their study was much higher, as compared to the two particles used in our study [41]. This might be attributed to the different particle coating. Even though higher ion release rates may be important for some silver nanoparticles and a number of other materials, the abovementioned ion release observed for the particles used in the present study will result in an only 3% decrease of the particle diameter and a change the total amount of delivered silver by less than 1%. These changes resulting from such minor dissolution phenomena are small and do not affect our main observations and conclusions. We want to point out, however, that this might not be the case for particles made from other materials with a higher rate of particle dissolution.

Even though there are a lot of caveats that need to be kept in mind when using in silico dosimetric calculations, these calculations can be used as a tool to get more accurate estimations of the real cellular dose. So far all published models have advantages and disadvantages and different fields of application. Especially when comparing different nanoparticles or different studies these calculations are important for proper interpretation. Just a direct measurement of delivered cellular dose of nanoparticles would be more correct and valuable.

## Conclusion

The presented particle dosimetry model 3DSDD can be used to calculate the delivered dose of nanoparticles in in vitro experiments, not only the basis of particle distribution, but also based on the diffusion coefficient measured by NTA. It accounts for 3D distribution of cells in in vitro cell culture dishes and is therefore suitable for differentiated cell models like Caco-2 cells, the most common model for the intestinal barrier, and HepaRG cells, a new and complex liver model. To encourage the use of dosimetry calculation software, our model can be downloaded from the Additional files 1, 2, 3, 4, 5, 6, 7 and 8.

## Materials and Methods

### Characterization of Caco-2 cell culture

The human colon adenocarcinoma cell line Caco-2 (European Collection of Cell Cultures (ECACC), Porton Down, UK) was maintained at 37 °C in a humidified atmosphere of 5% CO_2_ in Dulbecco’s modified Eagle’s medium (DMEM, PAN Biotech, Aidenbach, Germany) supplemented either with 1% (*v*/v) penicillin/streptomycin and 10% (v/v) heat-inactivated fetal calf serum (FCS) (PAA, Cölbe, Germany) for growth, or with 1% (v/v) ITS (insulin, transferrin, selenium) (PAA, Cölbe, Germany). For experiments with proliferating Caco-2 cells, cells were seeded in the desired multi-well plate and allowed to attach for 24 h. For differentiated Caco-2 cells, cells were cultivated for 21 days in the desired multi-well plate and cell culture medium was changed every 2 days. Day 1 was defined as the day when cells reached confluency. Cells were harvested on day 1, 6, 9, 16 and 21 after confluency by trypsinization and counted in a Neubauer counting chamber.

#### Protein content

Soluble cellular protein amounts were determined by the Bradford assay. Therefore, the harvested cells were freeze-thawed 3 times in liquid nitrogen and centrifuged for 10 min at 4 °C and 300 x g to remove aggregates. 20 μL of each sample was diluted into 200 μL Bradford staining solution, incubated for 30 min, and the absorption was measured at 595 nm using a Tecan plate reader (Männedorf, Switzerland). A calibration range between 5 and 70 μg/mL was chosen. Samples were diluted into the calibration range.

#### DNA content

DNA was isolated by phenol/chloroform extraction: the harvested cells were re-suspended in 250 μL Roti-phenol:chloroform:isoamylalcohol 25:24:1, shaken for 1 min, and centrifuged for 5 min at 300 x g. The aqueous phase was extracted by 200 μL Roti-chloroform:isoamylalcohol 24:1. The extracted DNA was precipitated using 1 mL ice-cold absolute ethanol and 150 μL 8 M ammoniumacetate at − 80 °C overnight. After precipitation, the samples were centrifuged for 30 min at 4 °C at 300 x g and the supernatants were discarded. Pellets were washed with 70% ice-cold ethanol and dried at 65 °C for 2 h. Dry pellets were dissolved in suitable amounts of TE buffer (10 mM Tris, 1 mM EDTA, pH 8) and DNA concentrations were determined photometrically using a Nanodrop spectrophotometer (Thermo Fisher Scientific, Waltham, MA, USA).

#### Cellular morphology

For determination of growth height, cells were cultivated until the day of interest. Medium was removed and the cells were stained with coomassie brilliant blue for 20 min. After that, cells were destained with H_2_O overnight. Plates were cut vertically to have a horizontal view into the well and on its wall. Growth height of the stained cells on the wall was measured with a sliding caliper.

#### Cross section cell size

Cellular morphology of confluent but undifferentiated as well as of differentiated Caco-2 cells was determined by confocal fluorescent microscopy (Leica SP5, Wetzlar, Germany) after staining with ActinRed 555 and ToPro-3 (Molecular probes, Thermo Fischer, Waltham, MA, USA). For measurements of the cell size a horizontal cut through the cell layer was chosen and the size of cells was measured using ImageJ software. At least 10 different images were analyzed resulting in at least 100 measured cells per image.

#### Cell cycle analysis

Cell cycle analysis was performed by propidium iodide (PI) staining. After trypsinization, cells were washed first with 700 μL PBS, then with 200 μL PBS. For fixation, 800 μL ice-cold ethanol was added. Pellets were incubated for 30 min on ice. After that, cells were washed again with PBS and stained with 150 μL PI staining solution (0.05 mg/mL PI, 10.6 U/mL RNAse A in PBS) for 15 min. Data for 10,000 cells per sample was collected with a flow cytometer. Fluorescence was determined (Ex: 488, Em: 585/40 nm) as a function of peak height vs. peak area to distinguish between cells with single DNA (G0/G1 phase) and with double DNA content (G2/M phase). The area in between was defined as synthesis phase (S phase). Relative cell fractions were calculated as mean values plus standard deviation of at least 4 replicates from at least 2 independent experiments. As inducer for cell cycle arrest, 100 nM colchicine was used.

### Characterization of nanoparticles and their dispersions

#### Nanoparticles

AgPURE silver nanoparticles contain 10% (w/w) silver and are stabilized with 4% (w/w) polyoxyethylene (20) sorbitan monolaurate (Tween 20) and 4% (w/w) polyoxyethylene glycerol trioleate (trade name Tagat TO). They were produced by Rent a Scientist GmbH (Regensburg, Germany). A comparable particle is used as reference material BAM001 by the Federal Institute for Materials Research and Testing (BAM) in Germany and reference material NM-300 from the Joint Research Centre (JRC) of the European Commission. [22, 1] AgPURE particles are about 15 nm in diameter, as determined by Small-angle X-ray scattering (SAXS) [3]. They were obtained and stored as stable dispersion. Stability was regularly monitored by DLS and NTA. Particles were diluted in cell culture medium as used for Caco-2 cultivation. The particles did not form a stable suspension in cell culture medium without FCS, but were stabilized in cell culture medium containing 10% FCS.

PAA-coated silver nanoparticles (AgPAA) were synthesized using silver nitrate and 1800 g/mol poly (acrylic acid) in a polyol process as originally described by Hu et al. and our previous work [15, 18, 26, 27]. These particles were about 6 nm in diameter, as determined by Small-angle X-ray scattering (SAXS) [18, 26, 27]. They were synthesized and stored as stable dispersion. Stability was regularly monitored by DLS and NTA. Particles were diluted in cell culture medium as used for Caco-2 cultivation containing 10% FCS and formed a stable dispersion.

#### Nanoparticle tracking analysis (NTA)

NTA measurements were performed with a NanoSight LM20 (NanoSight, Amesbury, United Kingdom), equipped with a sample chamber with a 640 nm red laser. The software used for capturing and analyzing the data was NTA 2.3 (NanoSight, Amesbury, United Kingdom). All measurements were performed at room temperature. The samples were injected into the sample chamber with sterile syringes. All samples were measured three times for 30 s with manual gain adjustments. Hydrodynamic diameter and diffusion coefficient were calculated.

#### Ion release

The amount of released silver ions from the nanoparticles was determined after incubation in cell culture medium with or without serum for 24 h under cell culture conditions in supernatant after centrifugation (1 h, 98,000 x g, 4 °C). Silver was analyzed by AAS using a graphite furnace AAS with a Transversely Heated Graphite Atomizer (THGA) and Zeeman background correction. The following temperature program was used: drying 10 s at 110 °C and 30 s at 130 °C, pyrolysis 20 s at 500 °C, atomization and analysis with a silver hollow-cathode lamp 2 s at 1700 °C and bake out 3 s at 2450 °C.

#### Determining effective density of particle dispersions - volumetric centrifugation

As the effective density does also depend on the suspension medium, all three used combinations of nanoparticles and cell culture medium were incubated under cell culture conditions (37 °C, humidified atmosphere and 5% CO_2_ for pH adjustment) for 24 h. Afterwards, samples were centrifuged in a PCV (packed cell volume) tube by a volumetric centrifugation method (Heraeus Multifuge 3SR+; 1 h, 2000 x g, 4 °C). The silver content in the supernatant was measured by AAS as described above, and the effective density of the nanoparticles was calculated from at least 6 replicates.

### In silico model

**Fig. 8:**
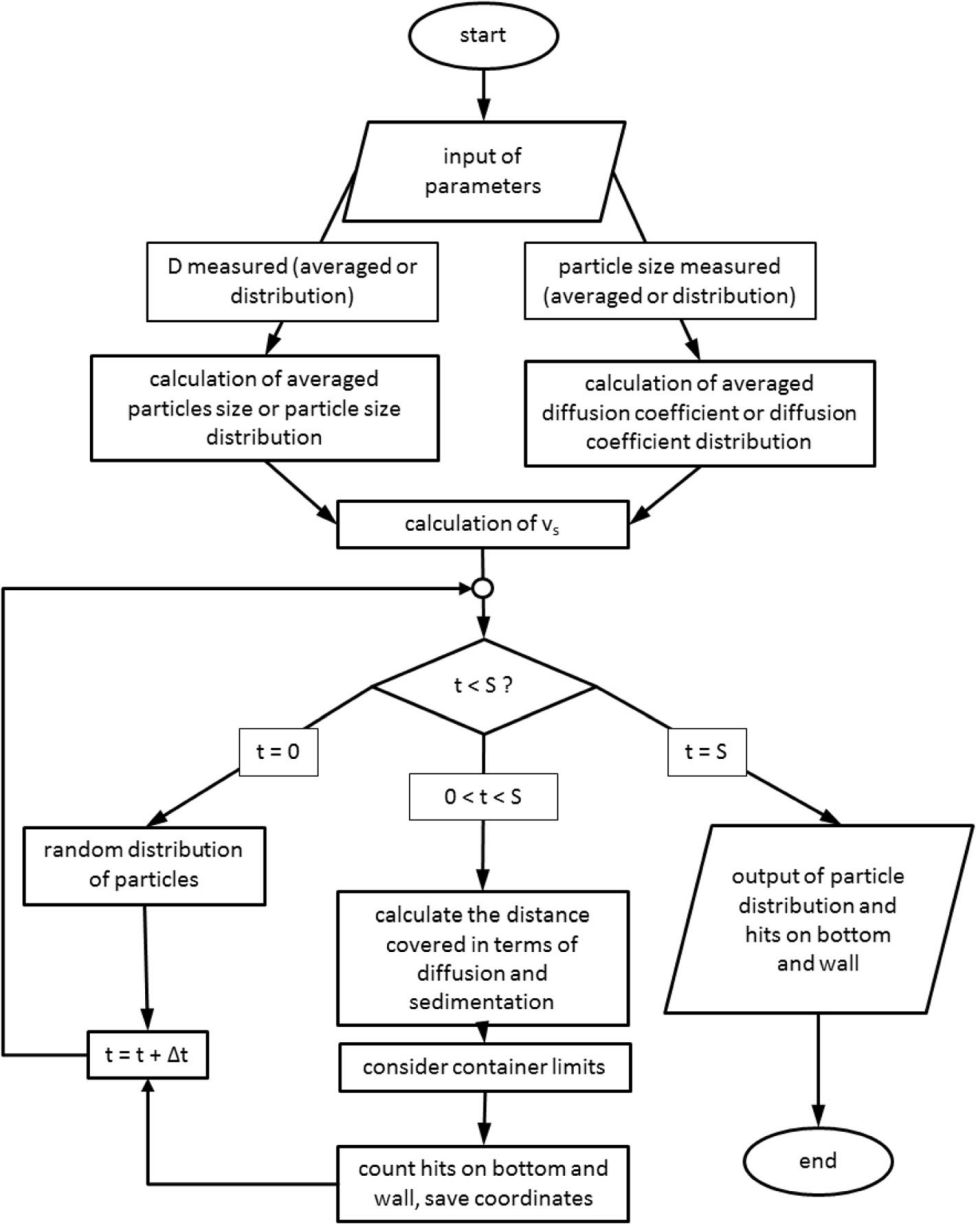
Workflow for in silico dosimetry calculation

While established models neglect the presence of cells on the sidelong wall of the dish [7, 13, 30, 32], the 3DSDD model calculates the particle dose delivered to the cells both on the wall and on the bottom, based on a Monte-Carlo algorithm. The model was implemented in Matlab® (MathWorks Inc.) by moving 10,000 non-interacting, spherical particles in terms of diffusion and sedimentation inside a cylinder with the dimensions of a well. The algorithm proceeds as depicted in Fig. 8. Initially the particles are placed randomly inside a cylinder with the same dimensions as the cell culture dish. During the simulation the particles are moved with regard to diffusion and sedimentation. The implementation of diffusion is based on the random walk of the particles in the fluid. Hence, the distance a particle moves in every time step Δt is a random number taken from the normal distribution Ɲ(0,2DΔt) [2]. This movement takes place in x-, y- and z-direction. Sedimentation is strongly affected by particle size, shape and density. Due to agglomeration, the hydrodynamic diameter of the particles increases. Furthermore, cell culture medium is entrapped in the interparticle space of the agglomerate. Hence, the density of the agglomerates, also called effective density, is lower than the density of the primary particle. Mostly, agglomerates are not composed of efficiently packed primary particles. This leads to a fractal structure which influences particle buoyancy. As a consequence, Stokes’s law has to be extended for the effective density *ρ*_*eff*_, the hydrodynamic diameter of the agglomerate *d*_*hyd*_ and the fractal dimension *DF* according to Sterling et al. [39].

$$ {v}_s=\frac{g\left({\rho}_{eff}-{\rho}_{fl}\right)}{18\eta }{d_{prim}}^{3- DF}{d_{hyd}}^{DF-1} $$

Here, *v*_*s*_ is the sedimentation velocity, *ρ*_*fl*_ is the fluid density, *d*_*prim*_ is the diameter of the primary particles and *g* is the gravitational constant.

The fractal dimension *DF* is described by the following relation

$$ DF=3+\frac{\log \left(1-\varepsilon \right)}{\log \left({d}_{hyd}\right)-\log \left({d}_{prim}\right)} $$

with the porosity *ε* being defined as

$$ \varepsilon =1-\frac{\rho_{eff}}{\rho_{prim}} $$

The necessary parameters are easily accessible. As model input the averaged diffusion coefficient or the diffusion coefficient distribution measured by NTA can be used. In case no NTA measurements are possible, the simulation can also be performed with the averaged particle size or the particle size distribution from which the diffusion coefficient is calculated using the Einstein-Smoluchowski relation:

$$ D=\frac{k_BT}{6\pi \eta r} $$

with *η* as the dynamic viscosity.

The other parameters needed for the input are easy to determine like effective density, medium viscosity, well dimensions, filling level, height of cell growth, medium temperature and medium density. For dose calculations the particles that reach the cells on the bottom or on the wall are counted as a hit and subsequently placed outside the cylinder. In case particles move outside the dish walls or the bottom due to diffusion or sedimentation the corresponding particle is placed back onto the dish wall. If it is located in a region with cell growth, the particle is counted as a hit and then placed outside the cylinder. Particles outside the cylinder are unable to move. Hence, a random walk back into the cylinder is suppressed. As soon as the simulation is over, the model outputs the delivered dose which is calculated for the bottom and the wall, respectively, by the following equation:

$$ {dose}_{del}=\frac{n}{N}\ast 100 $$

Here, *dose*_*del*_ is the delivered dose [%], *n* is the number of particles delivered, and *N* is the total number of particles.

### Measurement of density and viscosity of cell culture medium

The densities of the used cell culture medium were determined with an Anton Paar DSA 5000 M. This device works on the oscillating U-tube principle. The temperature was kept constant at 37 °C. The viscosity was measured by applying the Anton Paar MCR 302 with a temperature-controlled plate-plate device. The result of this method was the viscosity as a function of the shear stress. Therefore, the rheological behavior of all samples was determined. All samples showed Newtonian behavior.

### Characterization of the in silico 3DSDD model

For characterization of the influence of input parameters on the 3DSDD model outcome, the following calculations were performed exemplarily: with respect to the delivered cellular dose separated into cells on the bottom and wall the time, particle number, area of well bottom, hydrodynamic diameter of particles, effective density of particles and medium height was systematically varied. The following parameters were used for calculation: particle diameter 50 nm, effective density of nanoparticles 1.5 g/cm^3^, particle number 10000, temperature 37 °C, density of cell culture medium 1.0037 g/cm^3^, viscosity of cell culture medium 0.725 mPa s, height of cell growth on the cell culture dish wall 0.54 cm, surface area of cell culture dish bottom 0.34 cm^2^ (96-well plate), cylindrical shape of the cell culture dish, height of medium level in well 0.57 cm (corresponds to about 200 μL in 96-well, 570 μL in 48-well, 2.2 mL in 12-well and 5.5 mL in 6-well) and incubation time 24 h.

In addition, particle hits on the cell culture dish wall were studied in detail (under conditions disregarding cell growth) by changing the input parameters particle size and stickiness of particles at the wall. The following parameters were used for calculation: particle diameter 50 nm, effective density of nanoparticles 1.5 g/cm^3^, particle number 10000, temperature 37 °C, density of cell culture medium 1.0037 g/cm^3^, viscosity of cell culture medium 0.725 mPa s, surface area of cell culture dish bottom 0.34 cm^2^ (96-well plate), cylindrical shape of the cell culture dish, height of medium level in well 0.88 cm and incubation time 48 h.

### Model verification

For model verification two different approaches were chosen, a comparison with other already published in silico models, and experimental quantification of silver in horizontal slides of the fluid column of a nanoparticle dispersion.

#### Comparison with published in silico models

For comparison with other models from the literature, the In vitro Sedimentation, Diffusion and Dosimetry model (ISDD) from Hinderliter et al. and the Distorted Grid model (DG) from DeLoid et al. were used [7, 13]. ISDD was kindly provided by Justin G. Teeguarden and used in combination with the effective density of nanoparticles in cell culture medium, as experimentally determined by a PCV-based volumetric centrifugation method (VCM) described by DeLoid et al. [6]. For comparing VCM-ISDD and 3DSDD, literature-based parameters for titanium dioxide nanoparticles were chosen form [6]. These parameters are listed in Table 1 [6]. For comparing DG and 3DSDD, literature-based parameters for cerium dioxide nanoparticles were chosen from [7]. These parameters are also listed in Table 1 [7]. The DG and the ISDD models both do not take into account the cells on the wall of the cell culture dish. Thus the 3DSDD was used either, for a better direct comparison with the previous models, disregarding the cells on the wall, or in its intended form considering the cells on the wall. The filling level of the dish and therefore the height of exposed cells on the side wall was 0.3 cm for comparison with the DG model, and 0.315 cm for comparison with the ISDD model. Measurements from a 96-well plate were used. In the DG and the ISDD model the option “sticky” was used (particles adhere to the bottom) and particle voids were neglected.

#### Comparison with experimental results

For comparison with experimental results, cryosections of nanoparticle dispersions in cell culture medium were prepared and analyzed as follows: nanoparticle dispersions in cell culture medium were incubated under cell culture conditions for 0 h, 24 h, or 1 week in vessels with approximately the same size as 96-well plate wells. After incubation, suspensions were snap-frozen at − 80 °C. Samples were then cut in horizontal sections at − 20 °C. The amount of silver in every section was analyzed by AAS after acidic digestion. The workflow is also depicted in Fig. 6a. Silver content was analyzed by AAS using a graphite furnace AAS with a Transversely Heated Graphite Atomizer (THGA) and Zeeman background correction. The following temperature program was used: drying 10 s at 110 °C and 30 s at 130 °C, pyrolysis 20 s at 500 °C, atomization and analysis with a silver hollow-cathode lamp 2 s at 1700 °C, and bake out 3 s at 2450 °C.

3DSDD calculations were done three times using the parameters given in Table 2 and the NTA-measured diffusion coefficient after 24 h per silver nanoparticle type. Since there are no cells on the wall of the silicone tube, the cells on the walls were neglected in these model calculations. To determine the dose in each slice the cylinder was discretized using horizontal slices, corresponding to the height of the cryoslices. In each horizontal slice the number of particles was counted after 0 h, 24 h and 1 week and subsequently converted into the dose. The 3DSDD model was run 3 times and mean and standard deviation were calculated and expressed in percentage.

#### Utilization of the model and analytical determination of cellular silver contents

The verified 3DSDD model was used to calculate the delivered dose for an in vitro experiment with differentiated Caco-2 cells. For the experimental part, Caco-2 cells were seeded in 12-well Transwell inserts and differentiated for 21 days with a medium change every 2 days. Cells were then incubated under cell culture conditions for 24 h with silver nanoparticles in cell culture medium (Dulbecco’s modified Eagle’s medium (DMEM)) containing 10% FCS, 100 IU/mL penicillin and 100 μg/mL streptomycin, or containing 1% insulin, transferrin and selenium (ITS). The incubation volume was set to 500 μL which equals a medium height of 4.5 mm in the insert. Therefore, areas of 1.12 cm^2^ on the bottom and 1.688 cm^2^ on the wall are covered with cell culture medium. Afterwards, cells were washed 3 times with PBS to remove nanoparticles that had not been taken up but stuck to the surface of the cell monolayer. To determine silver uptake into the cells, we used a combination of washing steps to remove loosely bound particles and subsequent element analysis: cells on the wall and bottom of the insert were harvested separately and subjected to acidic microwave digestion (concentrated HNO_3_ in an ETHOS microwave from MLS, Leutkirch, Germany). Silver content was analyzed via AAS as described above. For relating the cellular uptake of silver to the delivered dose of silver nanoparticles, the particle distribution was calculated using 3DSDD and the parameters from Table 2.

## Additional files

Additional file 1: Add_step. (M 4 kb)

Additional file 2: Distribution_processing. (M 2 kb)

Additional file 3: Plot_dose_results. (M 2 kb)

Additional file 4: README. (TXT 941 bytes)

Additional file 5: Table_writer. (M 1 kb)

Additional file 6: Three_DSDD (M 9 kb)

Additional file 7: Example_distribution. (XLSX 50 kb)

Additional file 8: Example_results. (XLSX 8 kb)

## Abbreviations

3DSDD: 3D-sedimentation-diffusion-dosimetry model
AAS: Atomic Absorption Spectrometry
BAM: Federal Institute for Materials Research and Testing
DG: Distorted Grid model
DMEM: Dulbecco’s modified Eagle’s medium
ECACC: European Collection of Cell Cultures
FCS: fetal calf serum
ISDD: In vitro Sedimentation, Diffusion and Dosimetry model
ITS: Insulin, transferrin, selenium
JRC: Joint Research Centre
NTA: Nanoparticle tracing analysis
PCV: Packed cell volume
SAXS: Small-angle X-ray scattering
THGA: Transversely Heated Graphite Atomizer
VCM: Volumetric Centrifugation Method
